# Comprehensive transcriptomic and proteomic analyses identify intracellular targets for myriocin to induce *Fusarium oxysporum* f. sp. *niveum* cell death

**DOI:** 10.1186/s12934-021-01560-z

**Published:** 2021-03-17

**Authors:** Hengxu Wang, Zhigang Wang, Weihui Xu, Kexin Wang

**Affiliations:** 1 College of Life Science and Agroforestry, Qiqihar University, Qiqihar, 161006 China; 2 Heilongjiang Provincial Technology Innovation Center of Agromicrobial Preparation Industrialization, Qiqihar, 161006 China

**Keywords:** *Fusarium oxysporum* f. sp. *niveum*, Myriocin, Expression of genes and proteins, Transcriptomic and proteomic, Intracellular molecules

## Abstract

**Background:**

Myriocin is a natural product with antifungal activity and is derived from *Bacillus amyloliquefaciens* LZN01. Our previous work demonstrated that myriocin can inhibit the growth of *Fusarium oxysporum* f. sp. *niveum* (Fon) by inducing membrane damage. In this study, the antifungal actions of myriocin against Fon were investigated with a focus on the effects of myriocin on intracellular molecules.

**Results:**

Analysis of DNA binding and fluorescence spectra demonstrated that myriocin can interact with dsDNA from Fon cells. The intracellular-targeted mechanism of action was also supported by transcriptomic and proteomic analyses; a total of 2238 common differentially expressed genes (DEGs) were identified. The DEGs were further verified by RT-qPCR. Most of the DEGs were assigned metabolism and genetic information processing functions and were enriched in ribosome biogenesis in eukaryotes pathway. The expression of some genes and proteins in ribosome biogenesis in eukaryotes pathway was affected by myriocin, primarily the genes controlled by the C6 zinc cluster transcription factor family and the NFYA transcription factor. Myriocin influenced the posttranscriptional processing of gene products by triggering the main RI (retained intron) events of novel alternative splicing; myriocin targeted key genes (*FOXG_09470*) or proteins (RIOK2) in ribosome biogenesis in eukaryotes pathway, resulting in disordered translation.

**Conclusions:**

In conclusion, myriocin was determined to exhibit activity against Fon by targeting intracellular molecules. The results of our study may help to elucidate the antifungal actions of myriocin against Fon.

**Supplementary Information:**

The online version contains supplementary material available at 10.1186/s12934-021-01560-z.

## Background

*Fusarium oxysporum* f. sp. *niveum* (Fon) is a pathogen of destructive soil-borne disease that can cause *Fusarium* wilt in watermelon and seriously threaten the production of watermelon [1]. The control of Fon depends on fungicides, such as carbendazim, myclobutanil and diniconazole [2]. However, these fungicides have high potential risks, as they can pollute the environment and threaten human health [3]. Biological control is one of the most promising methods for suppressing the growth of Fon owing to its advantages of environmental friendliness, safety, and sustainability [4]. For example, *Bacillus velezensis* Y6 and Y7 have demonstrated antimicrobial activity against *Ralstonia solanacearum* and *Fusarium oxysporum* [5]. In particular, some antimicrobial substances have been considered as potential substitutes for fungicides [6, 7]. For example, *Pseudomonas chlororaphis* subsp. *piscium* secretes phenazine-1-carboxamide, which can affect the activity of *Fusarium graminearum* and eventually cause cell death [8]. Bacillomycin D has been determined to be secreted by *B. amyloliquefaciens* SQR9, which can bind to the iron transport regulator Btr and subsequently modulate biofilm formation through the KinB-Spo0a-Sinl-SinR pathway to suppress the growth of *Fusarium oxysporum* [9].

Myriocin [(2S,3R,4R,6E)-2-amino-3,4-dihydroxy-2-(hydroxymethyl)-14-oxo-6-eicosenoic acid] is a natural product that is isolated from *Myriococcum albomyces*, *Melanconis flavovirens*, *Isaria sinclairii*, *Paecilomyces variotii* ATCC 74097 and *Bacillus amyloliquefaciens* LZN01 [10–12]. Many studies have demonstrated that myriocin exhibits antifungal and antibacterial properties [13] and is a serine palmitoyl transferase (SPT) inhibitor and immunosuppressant [14]. Previously, we observed that myriocin is one of the metabolites secreted from *Bacillus amyloliquefaciens* LZN01 [12]. This peptide exhibits strong *in vitro* antifungal activity by disrupting SPT activity and inducing membrane damage in Fon [15]. In addition, antibiotic studies against fungal pathogens indicate that the induction of membrane damage is not the only way that antibiotics kill cells but may also have various intracellular targets [16, 17]. In other words, few studies have investigated the intracellular response or molecular events governing myriocin-induced Fon cell death. Therefore, studies on intracellular targets are necessary to elucidate the antibiotic actions of myriocin.

To address this gap in the body of knowledge, in this study, various concentrations of myriocin (1.25 μg/mL (MIC), 2.5 μg/mL (2 MIC), 5 μg/mL (4 MIC) and 10 μg/mL (8 MIC)) were employed to investigate the intracellular response of Fon to myriocin, which may help to elucidate the antibiotic actions of myriocin.

## Results

### DNA-binding activity and fluorescence intensity

**Fig. 1:**
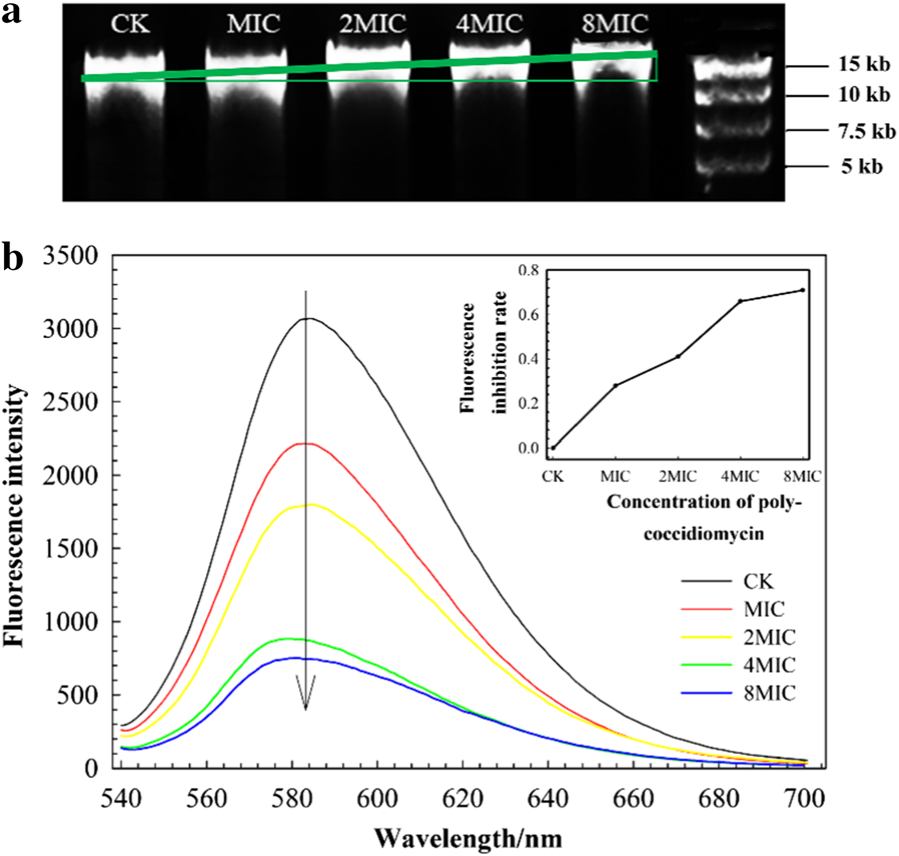
Analysis of the DNA-binding properties (**a**) and mode of action (**b**) of myriocin by gel retarding and fluorescence intensity assays, respectively. **a** Green right triangles show the electrophoretic mobility of Fon cell DNA bands. **b** The main diagram displays the changes in fluorescence intensity, and the small diagram shows the fluorescence inhibition rate of different concentrations of myriocin

The DNA binding ability of myriocin was examined by analyzing the electrophoretic mobility of Fon cell DNA bands at different concentrations of peptides to DNA on a 0.8% agarose gel. As shown in Fig. 1a, compared with 0 μg/mL (CK), with increasing concentrations of myriocin, such as the MIC (1.25 μg/mL) or higher concentrations (such as 8 MIC (10 μg/mL)), the migration of DNA bands gradually slowed in the gel. This result suggested that bound DNA had lower mobility, indicating the binding of myriocin to nucleic acids. To further demonstrate the possible mode of the binding of myriocin with DNA, the fluorescence spectra of the EB-DNA system in the presence of myriocin were studied. As shown in Fig. 1b, EB itself emitted strong fluorescence emission (CK), while a significant decrease in EB fluorescence intensity was observed with the addition of myriocin. The inhibition rate of fluorescence intensity increased with increases in myriocin concentration.

### Significant DEGs

At the mRNA level, the significant DEGs from CK_VS_MIC and CK_VS_8 MIC are shown in Additional file 1: Fig. S1a. In CK_VS_MIC, 2819 DEGs were detected, of which 1246 genes were upregulated and 1573 genes were downregulated. In CK_VS_8 MIC, 4143 DEGs were found, of which 1949 genes were upregulated and 2194 genes were downregulated. A total of 1275 genes of the 2238 common DEGs analyzed in both CK_VS_MIC and CK_VS_8 MIC were downregulated during myriocin treatment (Additional file 1: Fig. S1b). The results showed that the number of downregulated genes was greater than that of upregulated genes under myriocin treatment.

### Functional annotation and enrichment analysis of common DEGs

**Fig. 2:**
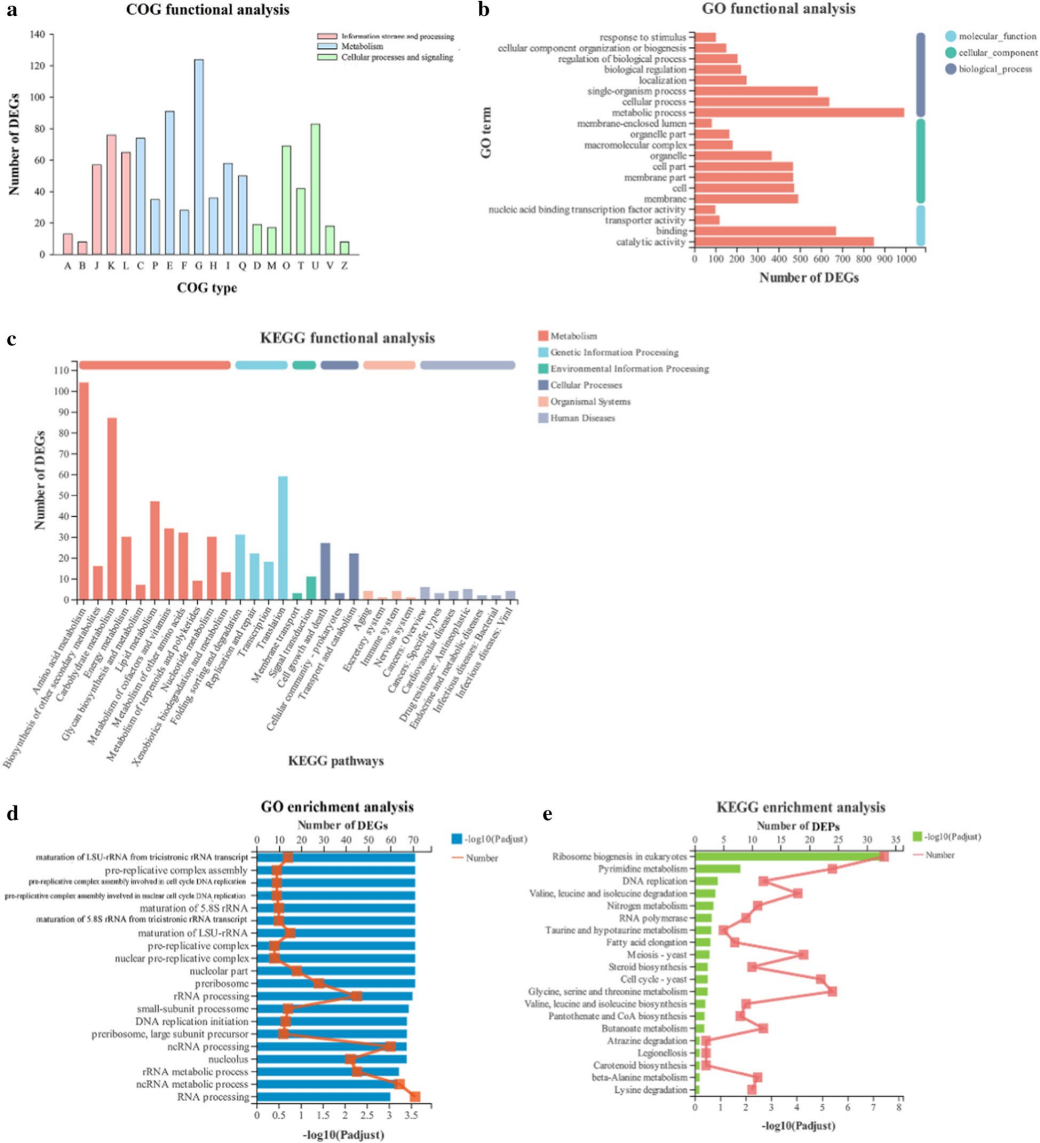
Functional annotation (**a**, **b** and **c**) and enrichment analysis (**d** and **e**) of common DEGs. The functional descriptions of the COG types are shown in Additional file 2: Table S3. GO categories and KEGG pathways significantly enriched (*P* < 0.05) are shown in the figure

The COG annotation showed that 2238 common DEGs were classified into 20 categories (Fig. 2a). Most of the common DEGs were assigned in the top five categories involving carbohydrate transport and metabolism (G), amino acid transport and metabolism (E), intracellular trafficking, secretion and vesicular transport (U), transcription (K), and energy production and conversion (C). Among these categories, three (G, E and C) belonged to metabolism, one (K) contained in information storage and processing, and one (U) ranged to cellular processes and signaling category. The common DEGs were classified according to GO functional analysis (Fig. 2b). Most of the common DEGs were categorized into biological process (BP) and molecular function (MF), which were primarily enriched in metabolic process, binding, catalytic activity, cellular process, and single-organism process. As shown in Fig. 2c, the assigned functions of DEGs covered six categories and involved thirty-one pathways. In terms of the number of DEGs, most DEGs were assigned metabolism and genetic information processing, and the number of distributed DEGs in amino acid metabolism and translation was higher than that in other pathways with the same function. The results showed that myriocin primarily affected the function of genes related to metabolism and genetic information processing.

The top 20 GO enrichment terms are shown in Fig. 2d, which involved 86 DEGs [75 upregulated genes and 11 downregulated genes (Additional file 2: Table S1)] that were primarily related to DNA and rRNA. A total of 346 common DEGs were assigned to 160 KEGG pathways. Fig. 2e shows that 168 common DEGs (92 upregulated genes and 76 downregulated genes) (Additional file 2: Table S2) were enriched in the top 20 KEGG pathways. Among these pathways, the top 10 KEGG enrichment pathways mainly belonged to genetic information processing (3 pathways) and metabolism (6 pathways) in function. Most of the DEGs were enriched in ribosome biogenesis in eukaryotes pathway, which was the most enriched pathway related to translation and belonged to the function of genetic information processing. The results demonstrated that the DEGs from BP were related to genetic information processing pathways and metabolism, which were significantly upregulated under myriocin exposure.

### Expression analysis of DEGs and DEPs in ribosome biogenesis in eukaryotes pathway

**Fig. 3:**
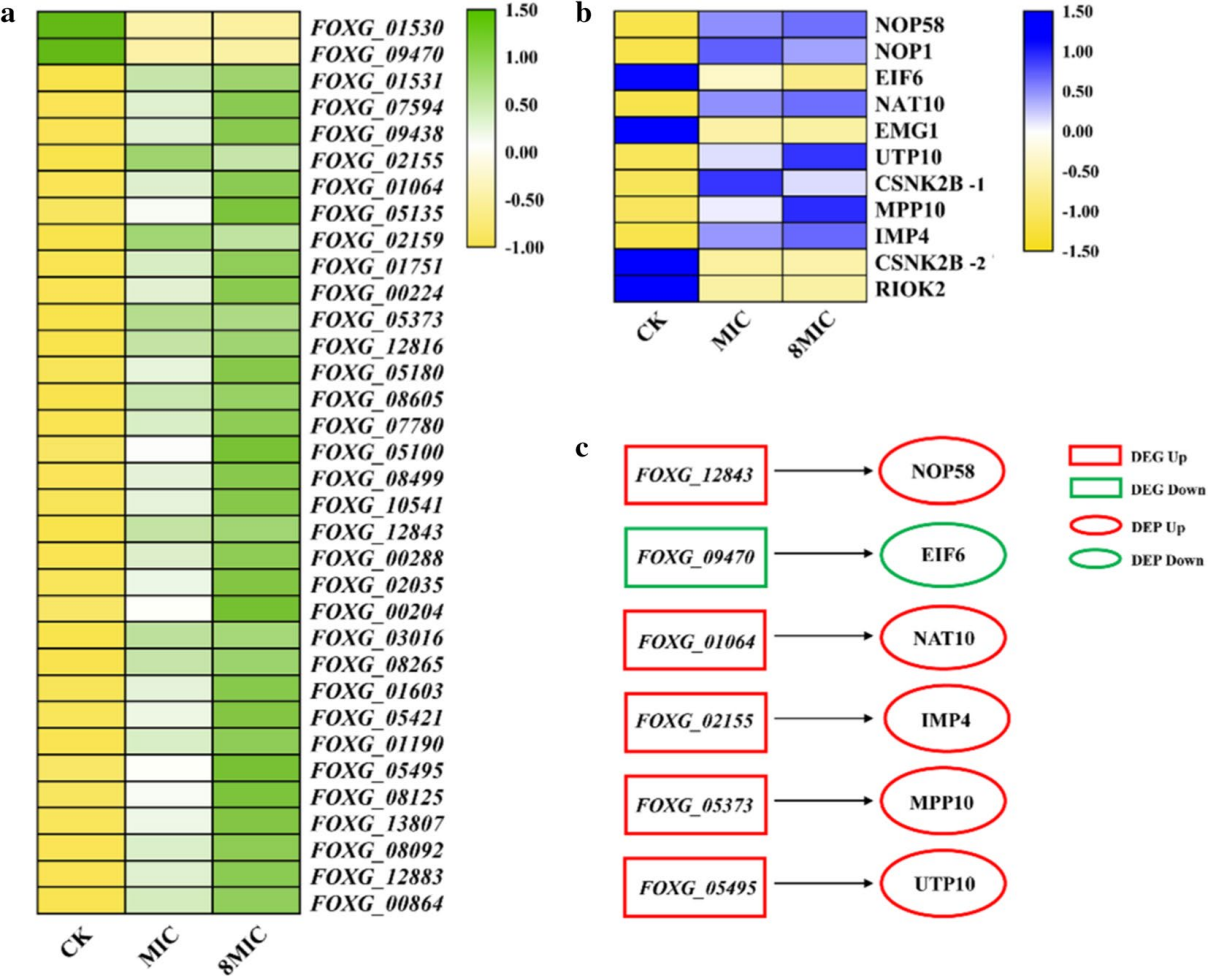
Heatmaps of the DEGs (**a**) and DEPs (**b**) and diagram of DEGs and corresponding DEG-encoded proteins in ribosome biogenesis in eukaryotes pathway. **a** Heatmap of DEGs. **b** Heatmap of DEPs. The colors indicate the expression level of the gene/protein [log10(TPM+1)]. The protein names are shown on the right, and KO names represent them. The correspondence of protein accession IDs and KO names are listed in Additional file 2: Table S4. **c** Each of the two linked frames represents a DEG and the DEG-encoded DEPs.

Expression analyses of DEGs and DEPs in ribosome biogenesis in eukaryotes pathway were performed. As shown in Fig. 3a, b, of the 34 common DEGs, 32 and 2 genes were significantly upregulated and downregulated, respectively, and of the 11 common DEPs, 7 and 4 proteins were significantly upregulated and downregulated under myriocin exposure, respectively. In addition, a total of 6 DEGs and 6 DEPs encoded by the DEGs in ribosome biogenesis in eukaryotes pathway were detected (Fig. 3c), and the trend of expression of the DEGs and DEPs encoded by the DEGs was similar. In other words, *FOXG_09470* and EIF6 encoded by *FOXG_09470* were significantly downregulated, and the other DEGs and DEPs encoded by the corresponding DEGs were upregulated, in myriocin-treated cells. These results indicated that some genes and proteins in ribosome biogenesis in eukaryotes pathway responded to myriocin.

**Fig. 4:**
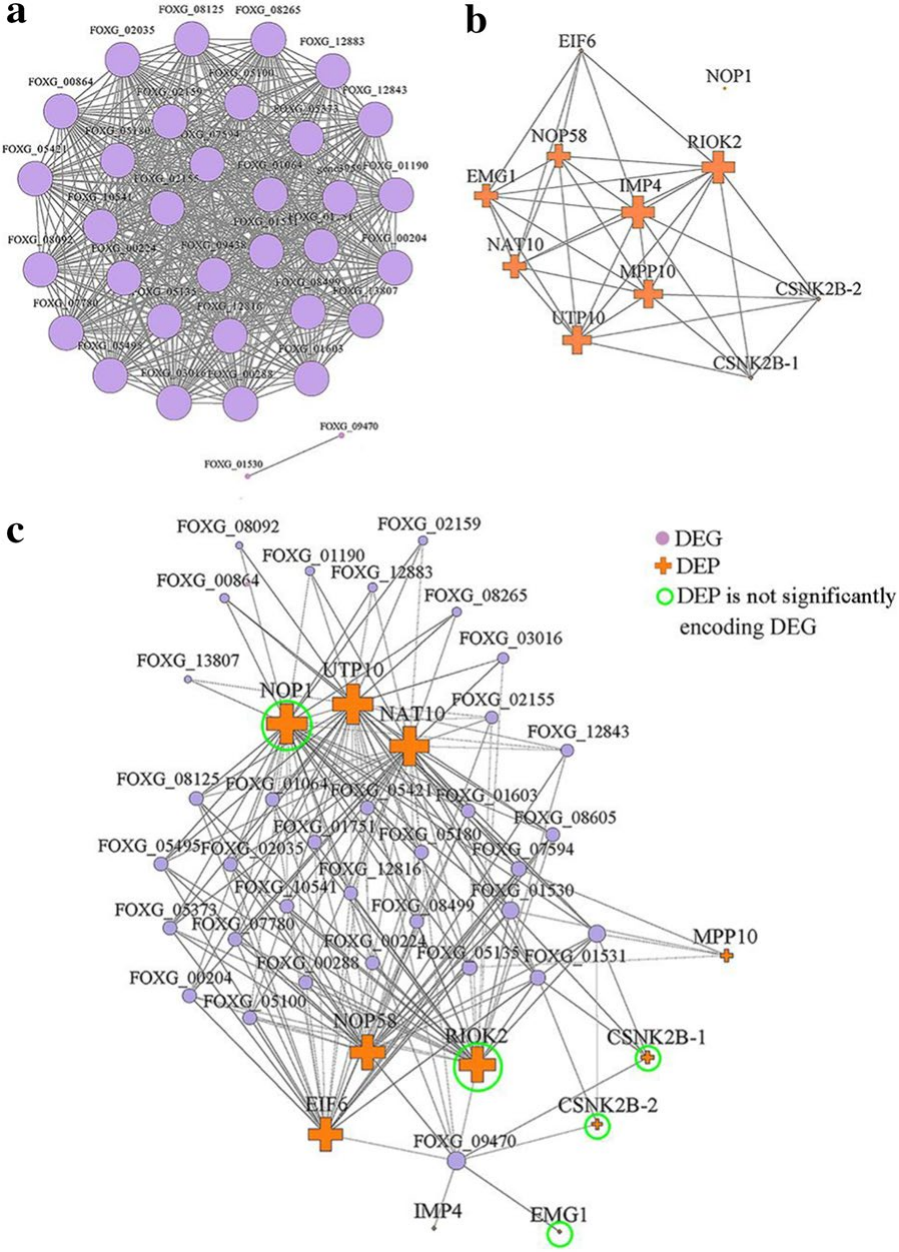
Correlation analysis of expression levels among DEGs (**a**), PPI network of DEPs (**b**) and correlation analysis network of expression levels among DEPs-DEGs (**c**). **a** and **b** Each node represents a DEG/DEP, and connecting lines represent the correlation of DEGs/DEPs. The larger the node is, the more important it is in the network. **c** Fully connected network of DEPs and DEGs. The nodes with the cross represent proteins, and the nodes with the circle represent genes. The larger the DEP node is, the more relevant it is to DEGs in the network. In addition, the nodes in the circle present DEPs, and the encoding gene of the protein is not a significant DEG

To further assess the relationship between DEGs and DEPs in ribosome biogenesis in eukaryotes pathway, correlated network analysis of DEG-DEG, DEP-DEP (PPI) and DEG-DEP was performed by using Cytoscape (v3.3.0). The correlation of the expression levels of 34 genes was divided into two parts. The first part consisted of 32 upregulated DEGs that had the same strong correlation of expression level among them (Fig. 4a). The second part consisted of 2 downregulated DEGs (*FOXG_01530* and *FOXG_09470*). The results indicated that 32 DEGs from ribosome biogenesis in eukaryotes pathway were correlated. In the PPI network, a total of 10 DEPs interacted. RIOK2 (atypical/RIO/RIO2 protein kinase) and IMP4 (hypothetical protein FOXG_05495) were correlated with the expression levels of the 9 DEPs (Fig. 4b). The degree centrality, closeness centrality and betweenness centrality values of RIOK2 and IMP4 were 1, 1 and 0.08 (Additional file 1: Fig. S2), respectively, and these values were higher for the two DEPs than for the other DEPs. Therefore, RIOK2 or IMP4 was the most closely correlated with other DEPs. To understand the relationship between DEGs and DEPs, a correlated network of 11 DEPs and 34 DEGs was constructed (Fig. 4c). At the protein level, hypothetical protein FOXG_02155 (UTP10) and hypothetical protein FOXG_18778 (NOP1) were associated with 34 DEGs and exhibited the most correlation with the expression of DEGs. The tRNA (Met) cytidine acetyltransferase (NAT10) and atypical/RIO/RIO2 protein kinase (RIOK2) were associated with 32 and 27 DEGs, respectively. At the mRNA level, *FOXG_09470* exhibited the most correlation with the expression of most DEPs. The expression level of *FOXG_09470* was significantly downregulated by *RNA-seq* (Fig. 3), and it was verified by RT-qPCR (Additional file 1: Fig. S3a). The results showed that the expression of DEGs and DEPs in ribosome biogenesis in eukaryotes pathway had a strong correlation, which indicated that myriocin affected the expression of one DEG/DEP and triggered a series of reactions related to it. In addition, the expression levels of 5 DEPs and corresponding DEG-encoded proteins were not consistent among such proteins as NOP1, RIOK2, CSNK2B-1, CSNK2B-2 and EMG1 (Fig. 4c). RIOK2 was the core protein in the PPI (Fig. 4b), which plays an important role in interacting with the DEP and DEG networks. However, the expression of *FOXG_08276*, which encodes RIOK2, was not significantly changed, as determined by *RNA-Seq* and RT-qPCR (Additional file 1: Fig. S3b), in myriocin-treated cells.

### Alternative splicing

Common types of alternative splicing (AS) events include retained introns (RIs), skipped exons (SEs), mutually exclusive exons (MEXs), alternative 5’ splice sites (A5SSs) and alternative 3’ splice sites (A3SSs) by using rMATS [18]. The analysis of novel AS in myriocin-treated Fon cells was performed using mRNA-Seq data. In CK_VS_MIC, a total of 1528 genes exhibited novel AS changes that were differentially expressed, including 1167 SE types, 126 MEX types and 235 RI types. Among these genes, 4 exhibiting SE, 15 exhibiting MEX and 36 exhibiting RI were significantly differentially expressed (Additional file 2: Table S5). In CK_VS_8 MIC, 1377 novel AS genes were differentially expressed, of which 1085, 112 and 207 novel AS genes belonged to the SE, MEX and RI types, respectively. Among these genes, 13 exhibiting SE, 11 exhibiting MEX and 36 exhibiting RI were found to be significantly differentially expressed (Additional file 2: Table S6). These results showed that most of the differentially expressed novel AS events belonged to the SE type, and most of the significant differentially expressed novel AS events belonged to the RI type in both CK_VS_MIC and CK_VS_8 MIC.

**Fig. 5:**
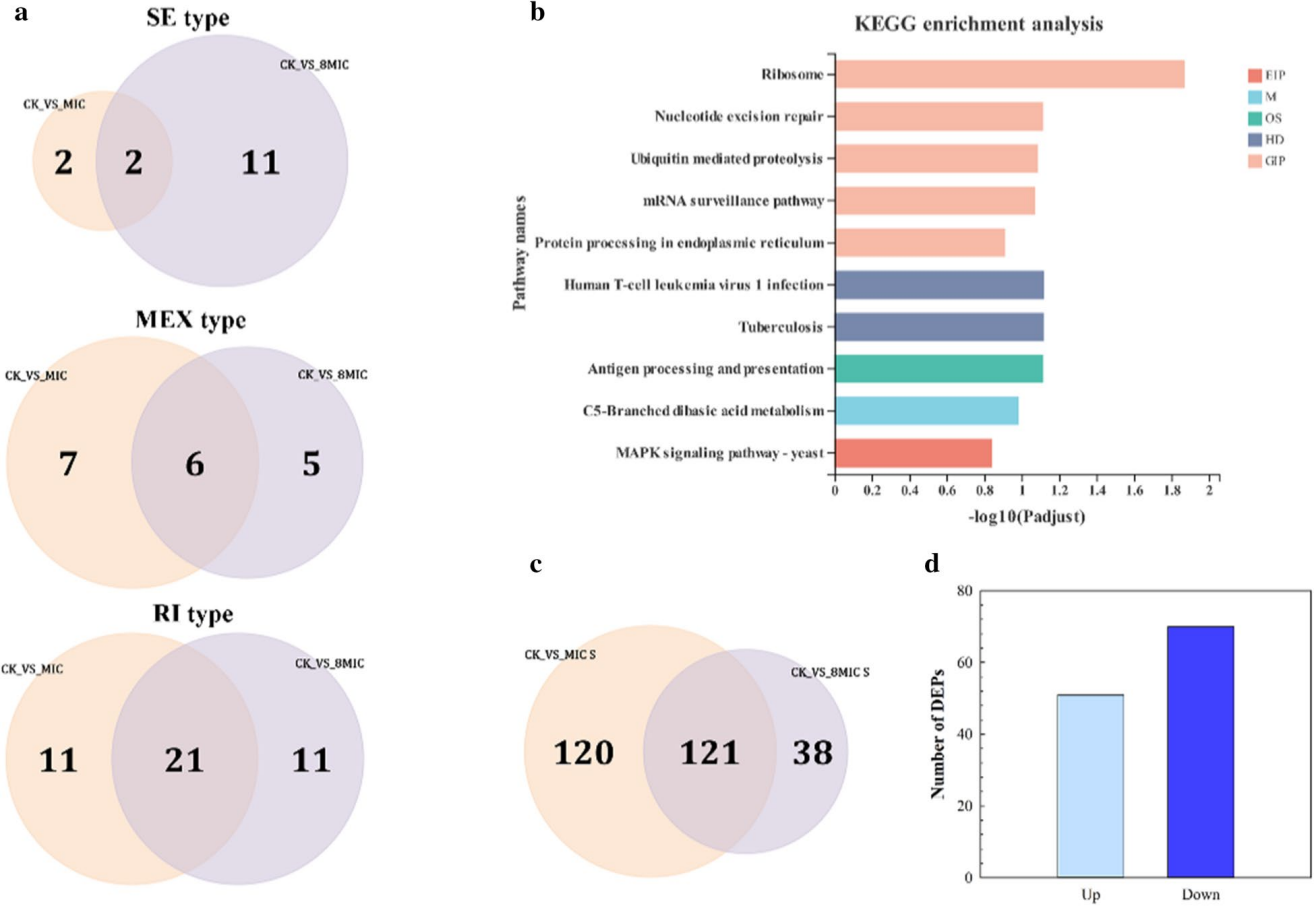
Venn diagram of AS type (**a**), functional enrichment of significant differentially expressed novel AS genes (**b**), Venn diagram (**c**) and number of DEPs (**d**). **a** Venn diagrams represent the common genes of significant novel AS events in CK1_VS_MIC and CK1_VS_8 MIC. **b** KEGG enrichment analysis of the common genes. **c** CK_VS_MIC S and CK_VS_8 MIC S represent the significant DEPs from the CK_VS_MIC and CK_VS_8 MIC treatments, respectively

At the mRNA level, significant differential changes in 29 novel AS genes were found, including 2 genes exhibiting SE, 6 exhibiting MEX and 21 exhibiting RI (Fig. 5a), which were primarily enriched in the ribosome, nucleotide excision repair and mRNA surveillance pathways (Fig. 5b). At the protein level, 303 DEPs in CK_VS_MIC and 277 DEPs in CK_VS_8 MIC (Additional file 2: Table S7) were influenced by differentially expressed genes in the AS. The 121 common DEPs between CK_VS_MIC and CK_VS_8 MIC (Fig. 5c) were significantly differentially expressed. Among these proteins, 51 DEPs were upregulated, and 70 DEPs were downregulated (Fig. 5d). Notably, the number of significant DEPs was greater than that of significant AS events (DEGs). These results suggested that myriocin could trigger the main RI events of novel AS, resulting in an increase in the number of differentially expressed genes and proteins.

### Transcription factor

**Fig. 6:**
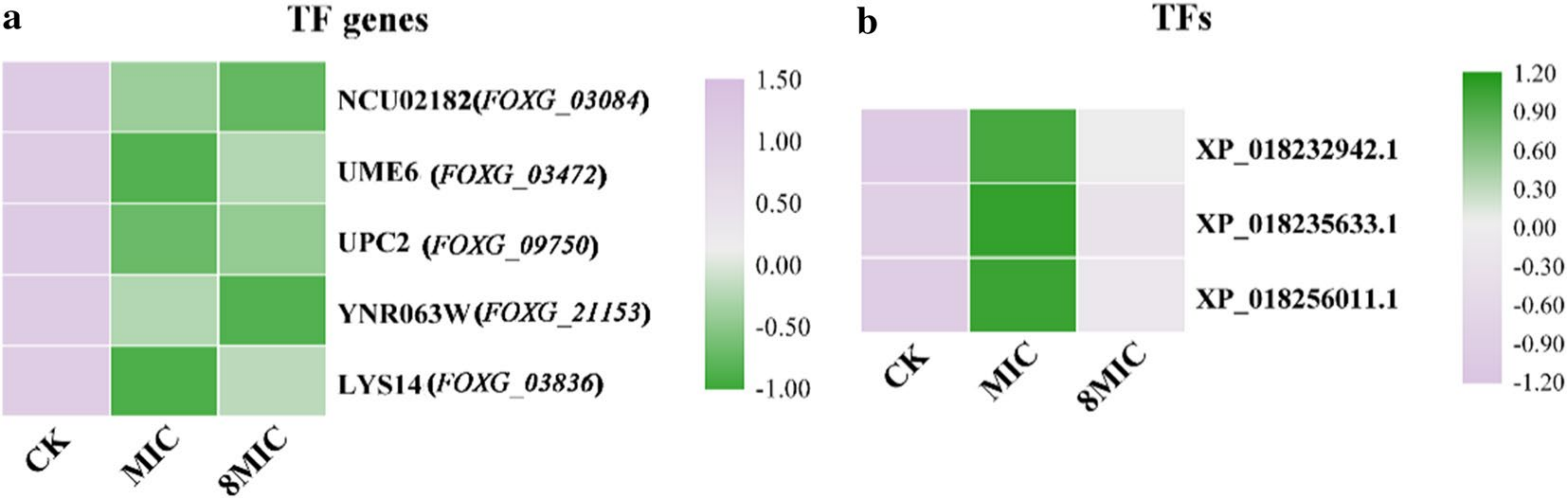
Expression level of controlled genes by TF (**a**) and TFs (**b**). **a** Controlled gene names by TF are shown on the right. **b** TF accession IDs were shown on the right. The TF accession IDs and controlled gene names by TF are listed in Additional file 2: Table S11

At the mRNA level, we found that 11 transcription factor (TF) target genes (a total of 11) and 9 TF target genes (a total of 13) were differentially expressed in CK_VS_MIC (Additional file 2: Table S8) and CK_VS_8 MIC (Additional file 2: Table S9), respectively. There were 5 common TFs (5 common controlled genes) in both CK_VS_MIC and CK_VS_8 MIC, including NCU02182, UME6, UPC2, YNR063W and LYS14 (Additional file 2: Table S10). Interestingly, the NCU02182, UME6, YNR063W, UPC2 and LYS14 target genes were significantly downregulated (Fig. 6a, Additional file 2: Fig. S4) by *RNA-Seq* and RT-qPCR. The target DEG of NCU02182 was *FOXG_03084,* whose main function was DNA binding (GO:0003677), as determined in GO annotation. The target DEGs of UME6, YNR063W, UPC2 and LYS14 were *FOXG_03472*, *FOXG_21153*, *FOXG_09750* and *FOXG_03836*, respectively. The main functions of the 4 DEGs involved regulation of transcription from RNA polymerase II promoter factor (GO:0006357), nucleus (GO:0005634), RNA polymerase II transcription factor activity, sequence-specific DNA binding (GO:0000981) and zinc ion binding (GO:0008270), as determined by GO annotation (Additional file 2: Table S13). The results showed that myriocin can induce downregulation of target genes of TF and may affect gene functions.

In addition, 3 differentially expressed TFs (Additional file 2: Table S11) were found at the protein level, including nuclear transcription factor Y, alpha, pH-response transcription factor pacC/RIM101, and transcription factor IWS1. The 3 TFs were upregulated (Fig. 6b), especially HAP2 (nuclear transcription factor Y, alpha, protein ID: XP_018232942.1), a member of the heteromeric CCAAT-binding factor family, which presented significant differential expression in myriocin-treated cells. The results of this analysis showed that the expression of TFs at the protein level varied in response to myriocin treatment.

### Molecular docking

**Fig. 7:**
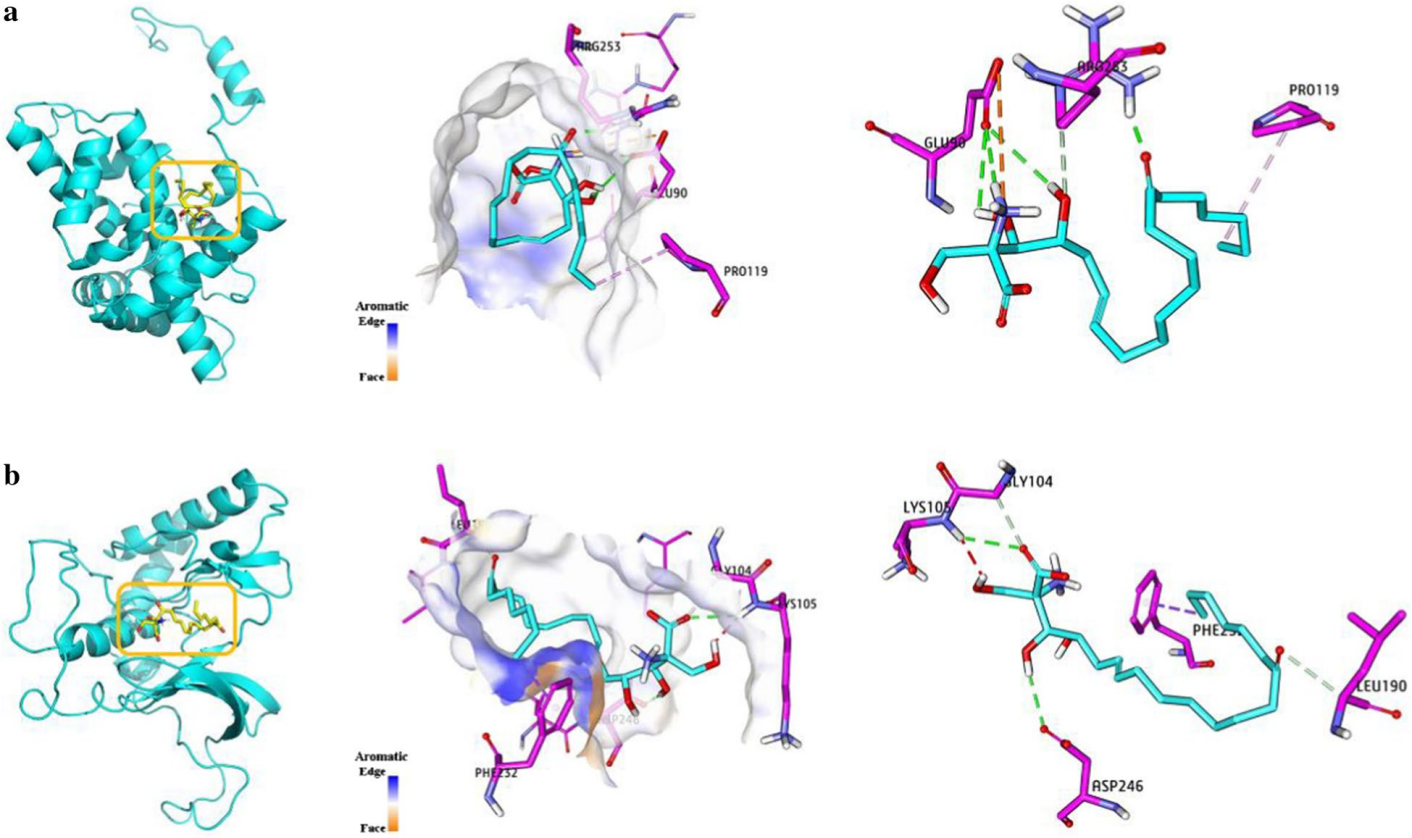
Molecular docking model of myriocin-NFYA/RIOK2. **a** and **b** represent the models of myriocin-NFYA and myriocin-RIOK2, respectively. From left to right, ribbons, surfaces and detailed binding diagrams are shown. Left: rectangular boxes represent internal docking areas. Middle: detailed internal docking areas (the surface color of proteins is classified by aromaticity). Right: detailed the bonding of myriocin-NFYA/RIOK2. GLU90, ARG253, PRO119, LYS105, ASP246, GLY104, LEU190, PHE232, PRO195 and ILE235 are amino acid residues. Hydrogen bonds are shown in green dashed lines. Electrostatic are shown in orange dashed line. Hydrophobics are shown in pink dashed line

To clarify the mode of action between myriocin and NFYA or RIOK2 at the molecular level, micromolecular myriocin was docked to NFYA and RIOK2. The affinities of the receptors were -5.5 and -5.0 kcal/mol, respectively. The combination patterns of myriocin-NFYA/RIOK2 are shown in Fig. 7. Myriocin bound to the active cavity of the NFYA (Fig. 7A) and RIOK2 (Fig. 7b) proteins. The two combinations matched well, which provided a useful foundation for further analysis of the interactions between myriocin and NFYA/RIOK2. The detailed interactions are presented in Additional file 2: Table S14. The acting forces consisted of 5 hydrogen bonds, 1 electrostatic interaction and 1 hydrophobic interaction in the combination of myriocin-NFYA. There were 4 hydrogen bonds and 3 hydrophobic interactions in the combination of myriocin-RIOK2. The number of hydrogen bonds was the highest, and it was the strongest force in the two combinations. These results indicated that myriocin could interact with NFYA and RIOK2 and form a stable complex [19], possibly explaining why NFYA and RIOK2 function was inhibited.

## Discussion

Some studies found that myriocin affected fungal activity by decreasing the content of sphingolipids (SPLs) [20] and inhibited hyphal growth and differentiation [21]. Our previous results indicated that myriocin induced membrane damage in Fon [15]. Few studies have investigated intracellular targets for myriocin to induce Fon cell death. In the present study, we focused on identifying intracellular targets for myriocin to induce Fon cell death by combined transcriptomic and proteomics analysis.

The interaction of myriocin with Fon cell DNA was examined first by gel retardation experiments. The results showed that myriocin decreased the migration of Fon DNA (Fig. 1a). This result indicated that myriocin had the ability to bind to Fon DNA [22]. Changes in fluorescence intensity can be used as evidence of the interaction between DNA and small molecules [23]. In the present study, EB fluorescence intensity was decreased with the addition of myriocin compared to CK (Fig. 1b), which could be attributed to the intercalation of myriocin in the double helix of DNA molecules. This finding indicated that the intercalated EB molecules on dsDNA could be replaced partially or fully by myriocin, which accounted for the decrease in fluorescence intensity [24]. Thus, it could be concluded that the binding of myriocin with dsDNA occurred primarily by the intercalation mode, which accounted for the decrease in the migration rate of the myriocin-DNA bound complex [23, 25].

As shown in Fig. S1, the number of downregulated DEGs were greater than that of upregulated DEGs. It implied that myriocin had a negative influence on mRNA level in Fon, which was consistent with the influence on Fon membrane in our previous study [15]. The number, function and enrichment of common DEGs are shown in Fig. 2. Most of the DEGs were classified to the category of biological process and assigned to metabolism and genetic information processing in KEGG function. Many of the DEGs were enriched in ribosome biogenesis in eukaryotes pathway (Fig. 2e), which belonged to genetic information processing in KEGG functional analysis. Genes from genetic information processing play dominant roles in transcription [26]. These results indicated that myriocin affected the function of genetic information processing in Fon cells, especially gene expression in ribosome biogenesis in eukaryotes pathway.

Transcription factors (TFs) play critical roles in organisms by regulating gene expression in various cellular processes [27] and play a key role in cell functions and determining responses to different environments [28]. We found that the genes controlled by NCU02182, UME6, YNR063W, UPC2 and LYS14 were significantly downregulated at the mRNA level (Fig. 6, Additional file 2: Table S4). The functions of genes controlled by the abovementioned TFs included DNA binding [29], regulation of transcription from the RNA polymerase II promoter factor [30], nucleus [31], RNA polymerase II transcription factor activity, sequence-specific DNA binding [32] and zinc ion binding [33]. It implied that expressions of genes controlled by the abovementioned TFs were affected by myriocin and may be cause disorder in functions. In addition, except for NCU02182, which belonged to the tryptophan cluster factor class, the other genes belonged to the C6 zinc cluster factor class. The 82 Zn (II) 2Cys6 DNA binding proteins, which are members of the C6 zinc cluster transcription factor family, are restricted to the fungal kingdom [34]. C6 zinc cluster transcription factors are in the pathogenic lifestyle of fungi and are not observed in nonpathogenic yeasts [35]. Butler et al. [36] reported that zinc-finger transcription factors were enriched in pathogenic *Candida* species and played key roles in adaptation to changes in the environment. The present study indicated that Fon is a pathogenic fungus that causes *Fusarium* wilt in watermelon. These results implied that myriocin could decrease the expression of genes controlled by C6 zinc cluster transcription factors, which may cause a reduction in the pathogenicity of Fon. Nuclear transcription factor Y, alpha (NFYA) is a highly conserved transcription factor that binds to CCAAT motifs of promoter regions in a variety of DNA [37] and regulates the transcription of numerous genes in eukaryotes [38]. NFYA was a significantly upregulated DEP at the protein level in myriocin-treated cells (Fig. 6b) and had a stable interaction with myriocin, which may cause abnormal binding to DNA [39].

Alternative splicing (AS) is an essential posttranscriptional process that increases transcriptomic and proteomic diversity and regulates gene expression in eukaryotes [40]. Different types of AS events exhibit different frequencies of occurrence in a variety of organisms; these AS events include SE and RI, which are the most universal events in animals and plants, respectively [41]. Although AS is common in higher plants and animals, its prevalence in fungi is mostly unknown [42]. In the present study, SE was the most prevalent event, and RI was the most significant event in Fon. AS events occur at pre-mRNA to increase the complexity of mRNA and then affect cellular complexity [43]. In this study, DEGs were primarily enriched in the ribosome, nucleotide excision repair and mRNA surveillance pathways. These pathways are primarily involved in translation, replication and repair steps of genetic information processing [44, 45]. Significant changes were observed in many DEPs encoded by these genes. These results implied that myriocin may be affect process of translation, replication and repair and result in increasing protein diversity, and then disorder of corresponding functions and Fon growth. Furthermore, in fungi, the involvement of AS in gene expression and its effect on cellularity and virulence is highly important [46]. The results suggested that myriocin may decrease the expression of some virulence genes by inducing AS events and subsequently reducing the pathogenic lifestyle [47], and also be used to support the decrease in expression of genes controlled by C6 zinc cluster transcription factors, which may cause a reduction in the pathogenicity of Fon [36].

In eukaryotes, ribosome biogenesis is an essential and fundamental process for cellular life [48]. The pathway is initiated by the transcription of a ribosomal RNA precursor (pre-rRNA) in the nucleolus followed by a series of processes to assemble the small and large ribosomal subunits. Eventually, the mature ribosomal subunits become functional for translation [49]. In this study, we found that the expression of some genes was altered by increases in AS events (Fig. 5) or interference with TFs in myriocin-treated Fon cells (Fig. 6). KEGG enrichment analysis showed that ribosome biogenesis in eukaryotes pathway responded strongly to myriocin. These results showed that non-normal expression of rRNA transcription and the transcriptional pathway could result in nonnormal translation in ribosomes [50, 51]. These results suggested that myriocin could cause non-normal expression of the ribosome biogenesis pathway and result in abnormal translation processes in Fon cells.

We found that the downregulation of *FOXG_09470* expression caused downregulation of EIF6 protein expression (Fig. 3c), and *FOXG_09470* correlated with the expression of other DEPs in the DEG-DEP network (Fig. 4c). RIOK2 was the most essential protein in the PPI network (Fig. 4b) and was a more important node in the DEP-DEG network (Fig. 4c). Therefore, *FOXG_09470* and RIOK2 are considered to play important roles in the interaction of genes and proteins. Combined analysis of transcriptomic and proteomic data suggested that myriocin could target key genes (e.g., *FOXG_09470*) [52] or proteins (e.g., RIOK2) [53] in ribosome biogenesis in eukaryotes pathway, change the expression of the genes or proteins and finally result in disordered translation. Notably, the gene encoding RIOK2 was not a significant DEG (Additional file 1: Fig. S3b), although RIOK2 was a significant DEP. However, RIOK2 was correlated with the expression of many genes in the DEP-DEG network. Molecular docking showed that myriocin interacted with RIOK2 through 4 hydrogen bonds and 3 hydrophobic interactions, which may affect the structure and expression of RIOK2 [15]. These results indicated that myriocin directly affected the expression of RIOK2 or indirectly changed the expression of associated genes. RIOK2 has been reported to be an important accessory factor in ribosome assembly [54]. The results of this study suggest that RIOK2 is an intracellular target for myriocin action.

## Conclusion

**Fig. 8:**
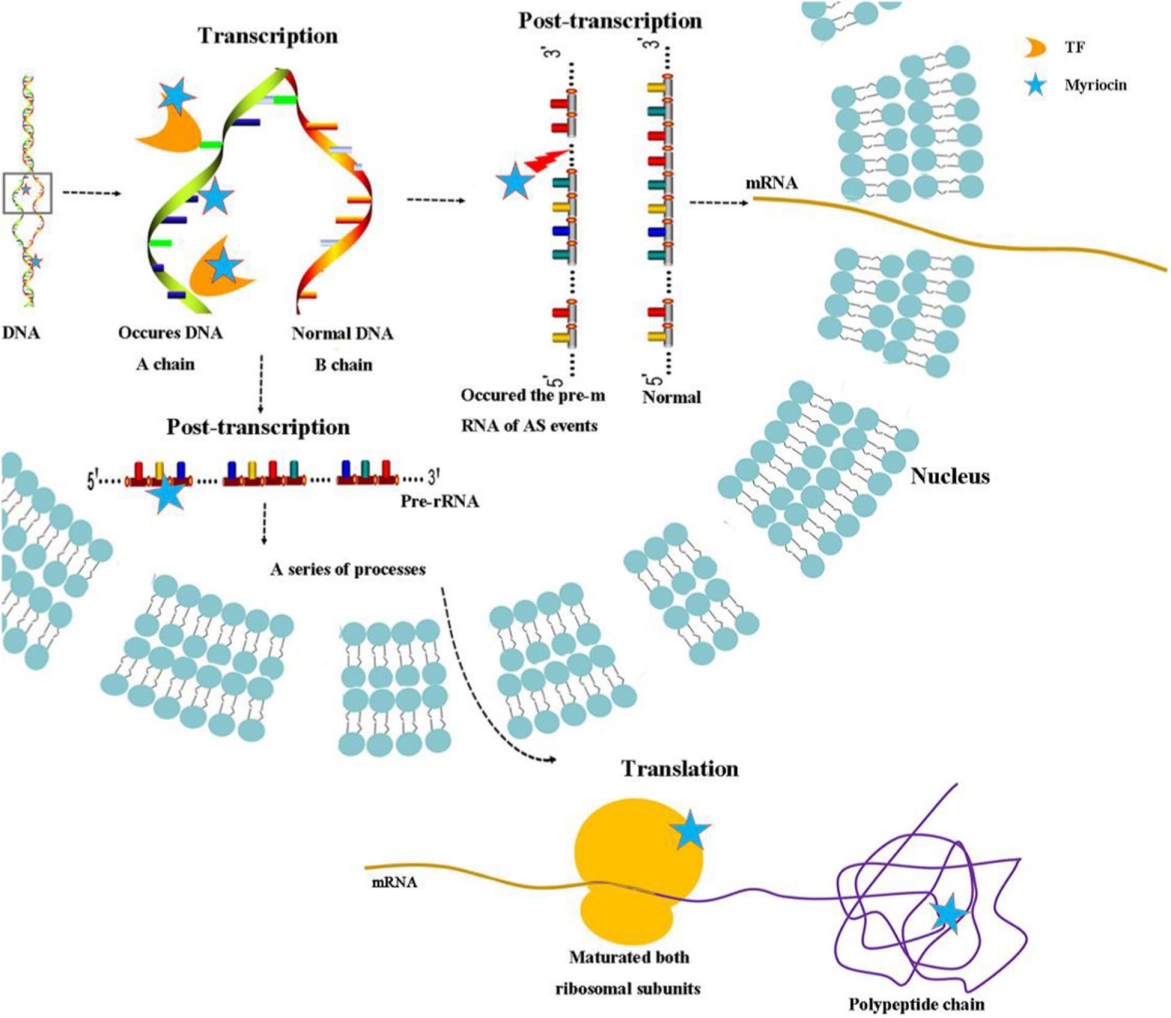
Conceptual model illustrating the intracellular-targeted mechanism of myriocin action on Fon

We have summarized the results of this study in a conceptual model (Fig. 8) that depicts the intracellular-targeted mode of action of myriocin against Fon. Myriocin bound to dsDNA of Fon by intercalation and decreased the migration rate of the myriocin-DNA bound complex. Myriocin changed the expression of genes controlled by the C6 zinc cluster transcription factor family and the NFYA transcription factor, which affected the normal process of transcription. Myriocin influenced the posttranscriptional processing of RNA by inducing alternative splicing events. Myriocin targeted key genes (*FOXG_09470*) or proteins (RIOK2) in ribosome biogenesis in eukaryotes pathway, which resulted in disordered translation. Combined with the findings of a previous study, we can cautiously conclude that the inhibitory activity of myriocin against Fon not only involves membrane damage but is also linked to interactions with intracellular targets, such as cell DNA, transcription factors, key genes (*FOXG_09470*) and proteins (RIOK2), in the ribosome biogenesis pathway. In conclusion, myriocin was determined to exhibit antifungal effects on Fon by targeting intracellular molecules.

## Materials and methods

### Cultivation of Fon

*Fusarium oxysporum* f. sp. *niveum* (Fon) used in this study was provided by the Laboratory of Microbial Ecology, Qiqihar University, China. This fungus was cultured in Petri dishes containing potato dextrose agar (PDA) medium for 5 days at 28°C to induce conidia (cells) [12]. Next, the dishes were soaked with sterile distilled water, the conidia were harvested from the culture surface, and a conidia concentration of 1.0×10^6^ colony forming units (CFUs)/mL was determined by a hemocytometer.

### Preparation of myriocin and distribution of treatment group

Myriocin was dissolved in DMSO and diluted to final concentrations of 1.25 μg/mL (MIC), 2.5 μg/mL (2 MIC), 5 μg/mL (4 MIC) and 10 μg/mL (8 MIC), which were considered treatment groups. The sample without myriocin was the control group (CK). The concentration of DMSO in solutions was less than or equal to 0.1% [55].

### DNA isolation, DNA-binding assay and fluorescence spectrum experiments

Genomic DNA was extracted from Fon according to the protocol supplied with the Ezup column fungi genomic DNA purification kit (B518259, Sangon Biotech, China), and the DNA was assessed using a Nanodrop 2000 spectrophotometer (OD_260_/OD_280_ = 1.7 ~1.9) and stored at -20°C until further analysis.

Assays of myriocin DNA binding activity were performed by gel mobility shift assays as described previously [56]. Briefly, 100 ng of Fon cell DNA was mixed with differential concentrations of myriocin in 20 μL of binding buffer (12.114 g/L Tris-HCl, 2.93 g/L EDTA buffer, 1.54 g/L dithiothreitol, 5% glycerinum, and pH 8.0). The mixtures were incubated at room temperature for 30 min. Samples were subjected to 0.8% agarose gel electrophoresis and stained with ethidium bromide.

As described previously [57], 20 μg/mL Fon DNA was mixed with 1 μg/mL EB and different concentrations of myriocin in 0.05 mol/L Tris–HCl buffer (pH 7.4). The concentrations of myriocin solutions were 0 μg/mL (CK), 1.25 μg/mL (1 MIC), 2.5 μg/mL (2 MIC), 5 μg/mL (4 MIC) and 10 μg/mL (8 MIC). Samples were incubated for 30 min, and the fluorescence spectrum was measured at excitation wavelengths (λex) = 260 nm, emission wavelengths (λem) = 540–700 nm and slit width = 5 nm by using a fluorescence spectrophotometer (F-7000, Hitachi, Japan).

The following formula was employed to calculate the inhibition rate of fluorescence intensity: Ri = 1-(*Ic*-*Ia*)/(*Ib*-*Ia*), where Ri is the inhibition rate of fluorescence intensity, *Ia* is the fluorescence intensity of 1 μg/mL EB, *Ib* is the fluorescence intensity of the CK sample and *Ic* is the fluorescence intensity of the MIC to 8 MIC samples [58].

### RNA-Seq

According to the previous results, myriocin at 1.25 μg/mL (MIC) and 10 μg/mL (8 MIC) was selected for the omics study. Fon conidia were treated with myriocin at final MIC and 8 MIC concentrations for 12 h, and the sample without myriocin was considered the control (CK). The mixtures were centrifuged at 8000 rpm for 10 min and subsequently frozen in liquid nitrogen immediately for RNA extraction and RNA sequencing. Three biological replicates were prepared for the control and treatment groups, respectively.

Total RNA was extracted from the different samples by using a Uniq-10 column TRIzol^®^ total RNA extraction kit (Invitrogen, USA) and treated with DNase I (TaKara, Japan) to remove the DNA. Three replicates per treatment were performed. The integrity and concentrations of the RNA were assessed using a 2100 Bioanalyzer (Agilent, USA) and a NanoDrop spectrophotometer (ND-2000, Thermo, USA). RNA sequencing libraries were constructed using high-quality total RNA samples (OD_260/230_ ≥ 2.0, OD_260/280_ = 1.8 ~ 2.2, RIN ≥ 6.5, 28S: 18S ≥ 1.0, > 2 μg). The RNA sequencing libraries were generated using the TruSeq^TM^ RNA Library Prep Kit for Illumina (San Diego, CA) following the manufacturer’s instructions, and index codes were added. First-strand cDNA synthesis was performed by using random hexamer primers and M-MuLV Reverse Transcriptase, and double-strand cDNA was synthesized by using a SuperScript Double-Strand cDNA Synthesis kit (Invitrogen, CA, USA). PCR amplification was performed using Phusion^®^ High-Fidelity DNA polymerase for 15 PCR cycles, and TBS380 (PicoGreen) was utilized to quantify the PCR amplification products. Next, the paired-end RNA sequencing libraries were sequenced on an Illumina HiSeq 4000 platform with 2 × 150-bp reads, and the raw paired-end reads were quality trimmed using SeqPrep (https://github.com/jstjohn/SeqPrep) and Sickle (https://github.com/najoshi/sickle) [59].

The raw sequencing data of RNA were uploaded to the Short Reads Archive of NCBI (https://www.ncbi.nlm.nih.gov/) with project number PRJNA643229. The clean reads were separately aligned to the Fusarium oxysporum reference genome using TopHat software, and the mapped results were subsequently queried against the database (ftp://ftp.ncbi.nlm.nih.gov/blast/executables/blast+/2.9.0/) using BLAST. The FPKM value was directly used to compare differences in gene expression among samples. Determination of the fold-change of transcripts and screening of differentially expressed genes (DEGs) were performed using the software package EdgeR. The criteria of significant difference expression were |log_2_ fold change| ≥ 1 and *p*-adjust ≤ 0.05. After screening DEGs, Gene Ontology (GO), Clusters of Orthologous Groups (COG) function and Kyoto Encyclopedia of Genes and Genomes (KEGG) pathway analyses were performed [15].

### Protein extraction and LC-MS/MS

The samples were prepared following the methods described in 2.4. Extraction buffer (1% SDS, 200 mM dithiothreitol, 50 mM Tris-HCl, and 1% protease inhibitor, pH 8.8) was utilized to extract proteins from samples. After centrifugation at 12000 rpm for 20 min at 4°C, the collected supernatants were precipitated with precooled acetone at -20°C overnight. Afterwards, the precipitate was obtained and washed two times with 90% precooled acetone. Next, lysis buffer (8 M urea, 1% SDS, and 1% protease inhibitor) was used to redissolve the protein precipitate, and the lysates were centrifuged; the protein concentration in the supernatant was quantified by using the bicinchoninic acid (BCA) method. Protein digestion was conducted following a standard procedure previously described by Morrin et al. [60]. Briefly, the appropriate amount of trichloroethyl phosphate was mixed with 150 μg of protein in each sample tube, and the mixtures were incubated at 37°C for 60 min. The sample was resuspended in 150 μL of buffer (100 mM TEAB). Trypsin was mixed with approximately 150 μg of protein for each sample (enzyme:protein, 1:50) and digested at 37°C overnight[60]. After trypsin digestion, the peptides were desalted using OASIS^®^ HLB μElution plates and vacuum dried, and the peptide concentrations were estimated using a peptide quantification kit (Thermo, Cat. 23275).

LC-MS/MS analysis was performed on a Q Exactive™ mass spectrometer (MS) (1200-6430A, Agilent, USA) [61]. The peptides were loaded onto a C18 reverse-phase column (75 μm×25 cm, Thermo, USA) with buffer A consisting of 2% acetonitrile and 0.1% formic acid and separated at a flow rate of 300 μL/min using a linear gradient of buffer B (80% acetonitrile and 0.1% formic acid). The peptides were subjected to nanoelectrospray ionization followed by tandem MS in Q Exactive (Thermo, USA) coupled to online UPLC. The resolution detection of the intact peptide segment in the orbitrap was 70,000. Peptides were selected for MS/MS by a high-energy collisional dissociation (HCD) setting of 20. Ion fragments were recognized in the orbitrap at a resolution of 17,500.

### Proteomics data analysis

The proteomics data were deposited to the ProteomeXchange Consortium (http://proteomiccentral.proteomexchange.org) via the iProX partner repository [62] with the dataset identifier PXD020176. MS/MS spectra were searched using ProteomeDiscover^TM^ Software 2.2 against the *Fusarium oxysporum* database (27347s, 20190328). Trypsin digestion was chosen with cleavage specificity allowing up to two missing cleavages. Carbamidomethyl of cysteine was designated as a fixed modification, and oxidation of methionine and protein N-terminal acetylation were assigned as variable modifications. A fold change > 1.2 or < 0.83 and a p-value < 0.05 served as the criteria of significant differential expression, which was used to screen differentially expressed proteins (DEPs) between the two samples. GO functional annotation and KEGG pathway enrichment analysis of DEPs were performed according to 2.4. A protein-protein interaction (PPI) network was constructed by using the STRING database (combined score ≥ 0.4).

### Analysis of combined transcriptomics and proteomic data

Cytoscape software (v3.3.0) was utilized to visualize the correlation analysis network of DEGs and DEPs. This software helped with selecting the major genes and target proteins in enrichment pathways and identifying expressed correlations among them. The Pearson correlation algorithm (correlation coefficients ≥ 0.5, p-value < 0.05) was employed to screen target proteins and major related genes from the combined proteomic and transcriptomic data.

### Quantitative real-time PCR validation

To verify the *RNA-Seq* results, 7 DEGs from the *RNA-Seq analysis* were selected, and quantitative real-time PCR (RT-qPCR) was conducted to confirm the expression changes of these 7 DEGs. In brief, total RNA was quantified at OD_260/OD280_ by using a SMA400 microspectrophotometer (Merinton, China). The purified RNA was reverse-transcribed into single-stranded cDNA by RevertAid Premium Reverse Transcriptase (EP0733, Thermo Scientific, USA) [63]. RT-qPCR was performed using a StepOne Plus Real-Time PCR instrument (ABI, Waltham, USA). Primers were used for RT-qPCR, which are shown in Additional file 2: Table S12, and the *18S rRNA* gene was used as the internal reference to which normalization of the mRNA abundance between samples was performed. Each treatment was repeated three times. The 2^−(ΔΔCt)^ method was utilized to calculate relative quantitative analysis.

### Molecular docking

In this study, the structure of myriocin was drawn by using ChemDraw Ultra 17.0 software. Next, the drawing was transformed into a 3D structure and optimized by using ChemBio3D Ultra 17.0 and MMFF94s. The 3D structure of NFYA (nuclear transcription factor Y, alpha) was downloaded from the Research Collaboratory for Structural Bioinformatics Protein Data Bank (the RCSB Data Bank) (www.rcsb.org), and NFYA (PDB ID: 4awl) was selected as the target protein in the docking. The 3D structure of RIOK2 (atypical/RIO/RIO2 protein linase) was obtained from a homology model through Swiss-Model. Both proteins and myriocin were converted to PDBQT format by using AutodockTools 1.5.6 [64, 65]. Autodock vina 1.1.2 was used to perform molecular docking of myriocin to the target protein [66]. The ‘exhaustiveness’ parameter was set to 20 to improve the accuracy of the calculation. Except for special instructions, other parameters were set to default values. Finally, conformation with the highest score was selected, and the results were analyzed by using the Discovery Studio 2019 client.

### Statistical analysis

SPSS 26.0 was used for statistical analysis of the data. The data are presented as the mean ± standard error from three biological replicates. The t-test was employed for statistical analysis. *P* < 0.05 was considered to be significant, and *P* < 0.01 was considered to be extremely significant.

## Supplementary Information

**Additional file 1: Figure S1.** Number (a) and venn diagram (b) of DEGs in CK_VS_MIC and CK_VS_8MIC. **Figure S2.** Distribution of the network center coefficient. **Figure S3.** Validation of *RNA-seq* data using RT-qPCR (*FOXG_09470* and *FOXG_08276*). **Figure S4.** Validation of *RNA-seq* data using RT-qPCR (*FOXG_03084*, *FOXG_03472*, *FOXG_09570*, *FOXG_21153* and *FOXG_03836*).

**Additional file 2: Table S1.** The names and regulation of 86 DEGs in GO enrichment analysis. **Table S2.** The names and regulation of 168 DEGs in KEGG enrichment analysis. **Table S3.** The information of COG. **Table S4.** The accession IDs and KO names of corresponding protein. **Table S5.** Differential alternative splicing events in CK_VS_MIC. **Table S6.** Differential alternative splicing events in CK_VS_8MIC. **Table S7.** Differentially expressed proteins and significant differentially expressed proteins in CK_VS_MIC and CK_VS_8MIC. **Table S8.** The information of differentially expressed genes by 11 TFs controlled in CK_VS_MIC. **Table S9.** The information of differentially expressed genes by 13 TFs controlled in CK_VS_8MIC. **Table S10.** The common TFs and its controlled common genes between CK_VS_MIC and CK_VS_8MIC. **Table S11.** The information of the differentially expressed 3 TFs. **Table S12.** Gene and primers used in RT-qPCR validation. **Table S13.** GO functional annotation of 4 DEGs. **Table S14.** Detailed interactions of myriocin-NFYA/RIOK2.

## Data Availability

All data generated or analysed during this study are included in this published article.
