# Supplementary material for: Comprehensive transcriptomic and proteomic analyses identify intracellular targets for myriocin to induce Fusarium oxysporum f. sp. niveum cell death
Source: Microb Cell Fact. 2021 Mar 17;20:69. doi: 10.1186/s12934-021-01560-z (PMC7968361; doi:10.1186/s12934-021-01560-z)
Supplement: Supplementary file 1 — Additional file 1: Figure S1. Number (a) and venn diagram (b) of DEGs in CK_VS_MIC and CK_VS_8MIC. Figure S2. Distribution of the network center coefficient. Figure S3. Validation of RNA-seq data using RT-qPCR (FOXG_09470 and FOXG_08276). Figure S4. Validation of RNA-seq data using RT-qPCR (FOXG_03084, FOXG_03472, FOXG_09570, FOXG_21153 and FOXG_03836). [file 12934_2021_1560_MOESM1_ESM.doc]

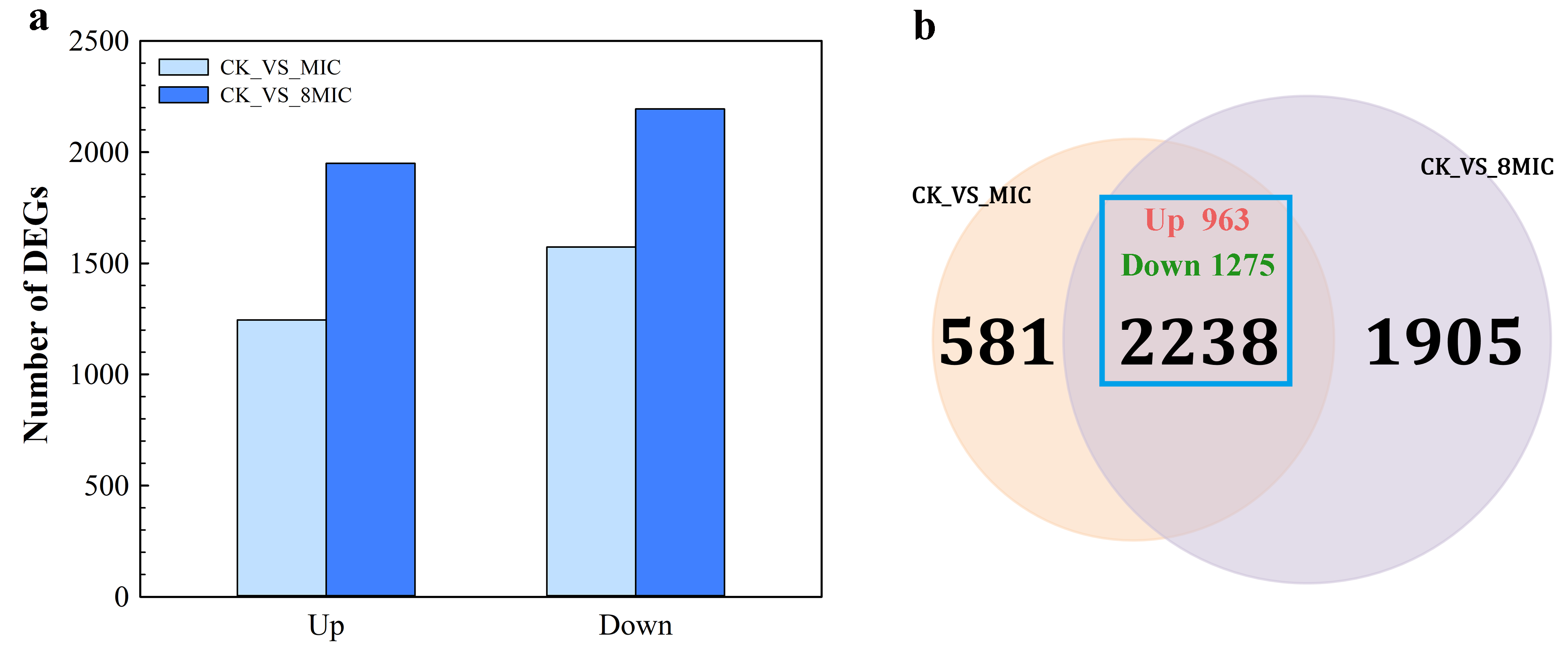


Figure S1. Number (a) and venn diagram (b) of DEGs in CK_VS_MIC and CK_VS_8MIC. **a** The differentially expressed genes (DEGs) at CK_VS_MIC and CK_VS_8MIC. A. The diagram to shown the number of DEGs upregulated and downregulated in different groups. **b** Venn diagram to shown the numebr of common DEGs between CK_VS_MIC and CK_VS_8MIC, and the expression of common DEGs.


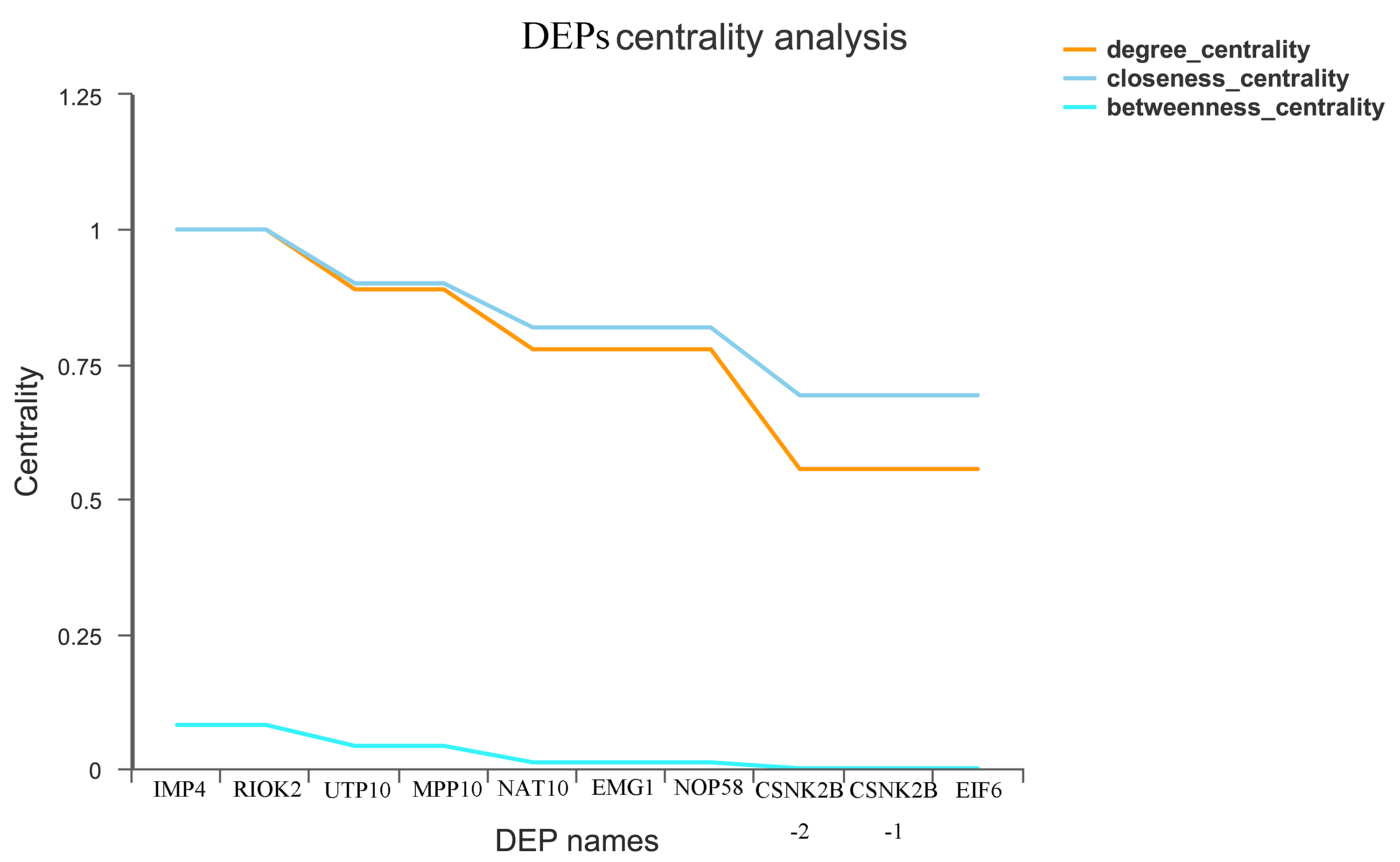


Figure S2. Distribution of the network center coefficient.


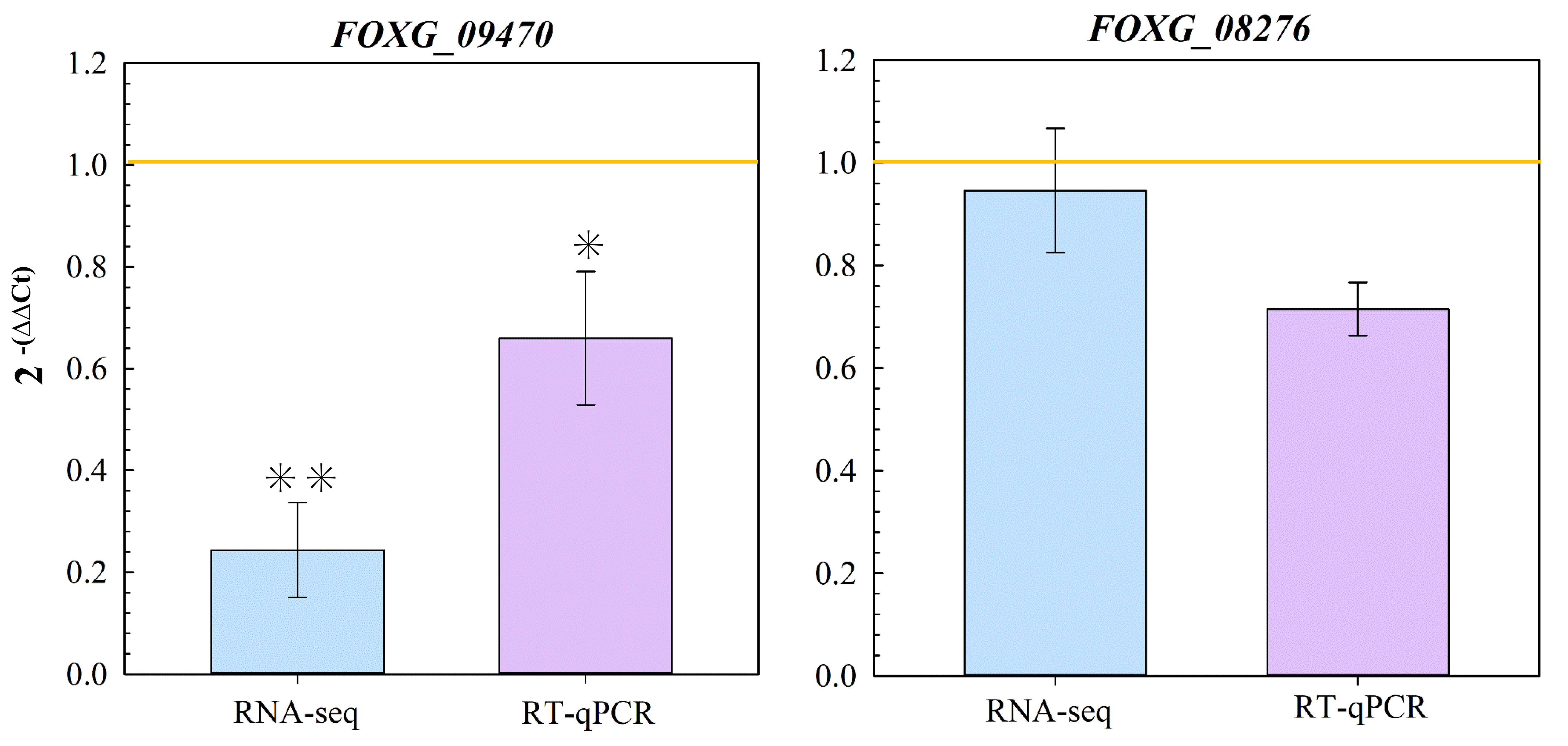


Figure S3. Validation of *RNA-seq* data by using RT-qPCR (*FOXG_09470* and *FOXG_08276*). Significant difference at *P*＜0.05(✳) , and Significant extremely difference at *P*＜0.01(✳✳).


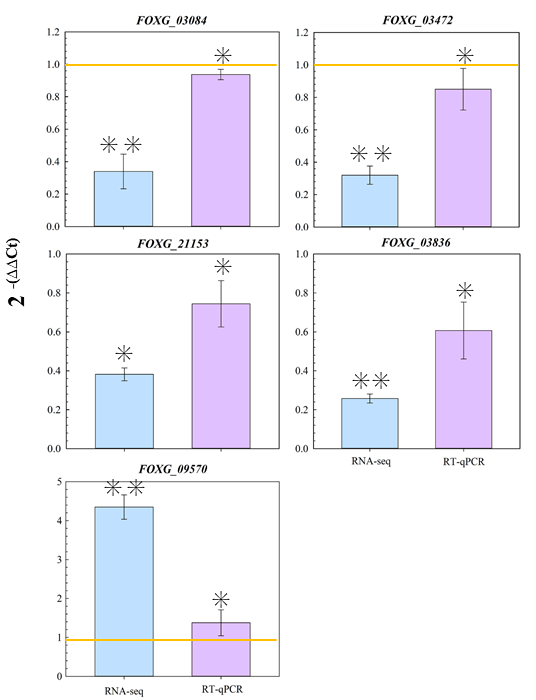


Figure S4. Validation of *RNA-seq* data by using RT-qPCR (*FOXG_03084*, *FOXG_03472*, *FOXG_09570*, *FOXG_21153* and *FOXG_03836*). Significant difference at *P*＜0.05(✳) , and significant extremely difference at *P*＜0.01(✳✳).
