# Supplementary material for: Comprehensive transcriptomic and proteomic analyses identify intracellular targets for myriocin to induce Fusarium oxysporum f. sp. niveum cell death
Source: Microb Cell Fact. 2021 Mar 17;20:69. doi: 10.1186/s12934-021-01560-z (PMC7968361; doi:10.1186/s12934-021-01560-z)
Supplement: Supplementary file 2 — Additional file 2: Table S1. The names and regulation of 86 DEGs in GO enrichment analysis. Table S2. The names and regulation of 168 DEGs in KEGG enrichment analysis. Table S3. The information of COG. Table S4. The accession IDs and KO names of corresponding protein. Table S5. Differential alternative splicing events in CK_VS_MIC. Table S6. Differential alternative splicing events in CK_VS_8MIC. Table S7. Differentially expressed proteins and significant differentially expressed proteins in CK_VS_MIC and CK_VS_8MIC. Table S8. The information of differentially expressed genes by 11 TFs controlled in CK_VS_MIC. Table S9. The information of differentially expressed genes by 13 TFs controlled in CK_VS_8MIC. Table S10. The common TFs and its controlled common genes between CK_VS_MIC and CK_VS_8MIC. Table S11. The information of the differentially expressed 3 TFs. Table S12. Gene and primers used in RT-qPCR validation. Table S13. GO functional annotation of 4 DEGs. Table S14. Detailed interactions of myriocin-NFYA/RIOK2. [file 12934_2021_1560_MOESM2_ESM.doc]

Table S1. The names and regulation of 86 DEGs in GO enrichment analysis.

| **Gene name** | **Regulate** | **Gene name** | **Regulate** | **Gene name** | **Regulate** |
| --- | --- | --- | --- | --- | --- |
| *FOXG_13642* | down | *FOXG_01196* | up | *FOXG_08090* | up |
| *FOXG_01935* | down | *FOXG_01751* | up | *FOXG_07670* | up |
| *FOXG_09470* | down | *FOXG_02309* | up |  |  |
| *FOXG_08361* | down | *FOXG_05373* | up |  |  |
| *FOXG_08286* | down | *FOXG_02046* | up |  |  |
| *FOXG_05357* | down | *FOXG_00686* | up |  |  |
| *FOXG_08458* | down | *FOXG_05180* | up |  |  |
| *FOXG_04214* | down | *FOXG_08605* | up |  |  |
| *FOXG_07665* | down | *FOXG_10590* | up |  |  |
| *FOXG_03094* | down | *FOXG_04156* | up |  |  |
| *FOXG_08675* | down | *FOXG_07780* | up |  |  |
| *FOXG_09455* | up | *FOXG_01433* | up |  |  |
| *FOXG_00984* | up | *FOXG_05100* | up |  |  |
| *FOXG_00048* | up | *FOXG_10431* | up |  |  |
| *FOXG_04073* | up | *FOXG_02230* | up |  |  |
| *FOXG_08091* | up | *FOXG_02004* | up |  |  |
| *FOXG_08363* | up | *FOXG_00085* | up |  |  |
| *FOXG_01452* | up | *FOXG_08293* | up |  |  |
| *FOXG_00552* | up | *FOXG_10250* | up |  |  |
| *FOXG_04212* | up | *FOXG_10541* | up |  |  |
| *FOXG_09436* | up | *FOXG_06406* | up |  |  |
| *FOXG_02034* | up | *FOXG_02367* | up |  |  |
| *FOXG_03416* | up | *FOXG_12843* | up |  |  |
| *FOXG_06295* | up | *FOXG_00288* | up |  |  |
| *FOXG_13843* | up | *FOXG_13830* | up |  |  |
| *FOXG_09517* | up | *FOXG_02035* | up |  |  |
| *FOXG_11106* | up | *FOXG_09304* | up |  |  |
| *FOXG_08313* | up | *FOXG_03016* | up |  |  |
| *FOXG_02155* | up | *FOXG_08265* | up |  |  |
| *FOXG_10293* | up | *FOXG_02200* | up |  |  |
| *FOXG_09477* | up | *FOXG_01603* | up |  |  |
| *FOXG_05693* | up | *FOXG_05421* | up |  |  |
| *FOXG_07755* | up | *FOXG_11626* | up |  |  |
| *FOXG_05135* | up | *FOXG_00216* | up |  |  |
| *FOXG_09435* | up | *FOXG_09190* | up |  |  |
| *FOXG_01578* | up | *FOXG_01019* | up |  |  |
| *FOXG_08314* | up | *FOXG_07993* | up |  |  |
| *FOXG_08549* | up | *FOXG_01494* | up |  |  |
| *FOXG_01853* | up | *FOXG_12795* | up |  |  |
| *FOXG_05440* | up | *FOXG_13886* | up |  |  |
| *FOXG_01697* | up | *FOXG_12684* | up |  |  |
| *FOXG_10487* | up | *FOXG_08092* | up |  |  |

Table S2. The names and regulation of 168 DEGs in KEGG enrichment analysis

| **Gene_name** | **Regulate** | **Gene_name** | **Regulate** | **Gene_name** | **Regulate** | **Gene_name** | **Regulate** |
| --- | --- | --- | --- | --- | --- | --- | --- |
| *FOXG_10934* | down | *FOXG_05288* | down | *FOXG_09685* | up | *FOXG_00224* | up |
| *FOXG_01626* | down | *FOXG_05881* | down | *FOXG_02109* | up | *FOXG_09389* | up |
| *FOXG_04181* | down | *FOXG_09201* | down | *FOXG_04166* | up | *FOXG_05373* | up |
| *FOXG_03110* | down | *FOXG_05564* | down | *FOXG_04072* | up | *FOXG_02046* | up |
| *FOXG_01530* | down | *FOXG_04394* | down | *FOXG_01590* | up | *FOXG_00800* | up |
| *FOXG_03313* | down | *FOXG_04715* | down | *FOXG_13032* | up | *FOXG_12816* | up |
| *FOXG_09337* | down | *FOXG_01425* | down | *FOXG_07773* | up | *FOXG_09260* | up |
| *FOXG_06001* | down | *FOXG_12736* | down | *FOXG_00512* | up | *FOXG_02257* | up |
| *FOXG_09470* | down | *FOXG_02425* | down | *FOXG_13921* | up | *FOXG_03363* | up |
| *FOXG_07961* | down | *FOXG_11134* | down | *FOXG_00048* | up | *FOXG_05180* | up |
| *FOXG_12862* | down | *FOXG_08547* | down | *FOXG_04073* | up | *FOXG_08605* | up |
| *FOXG_01161* | down | *FOXG_05182* | down | *FOXG_05355* | up | *FOXG_10590* | up |
| *FOXG_12350* | down | *FOXG_17352* | down | *FOXG_00335* | up | *FOXG_07780* | up |
| *FOXG_10933* | down | *FOXG_10711* | down | *FOXG_07594* | up | *FOXG_00214* | up |
| *FOXG_11436* | down | *FOXG_04303* | down | *FOXG_09116* | up | *FOXG_08668* | up |
| *FOXG_03192* | down | *FOXG_02429* | down | *FOXG_08091* | up | *FOXG_05100* | up |
| *FOXG_15247* | down | *FOXG_08094* | down | *FOXG_12887* | up | *FOXG_10431* | up |
| *FOXG_02077* | down | *FOXG_16922* | down | *FOXG_08578* | up | *FOXG_08499* | up |
| *FOXG_02078* | down | *FOXG_13316* | down | *FOXG_10540* | up | *FOXG_12313* | up |
| *FOXG_07800* | down | *FOXG_13669* | down | *FOXG_13311* | up | *FOXG_10541* | up |
| *FOXG_11520* | down | *FOXG_02267* | down | *FOXG_05142* | up | *FOXG_03715* | up |
| *FOXG_06404* | down | *FOXG_02315* | down | *FOXG_00552* | up | *FOXG_07921* | up |
| *FOXG_12668* | down | *FOXG_17736* | down | *FOXG_04212* | up | *FOXG_12843* | up |
| *FOXG_03312* | down | *FOXG_11025* | down | *FOXG_09212* | up | *FOXG_00962* | up |
| *FOXG_00374* | down | *FOXG_00088* | down | *FOXG_04298* | up | *FOXG_00288* | up |
| *FOXG_08976* | down | *FOXG_03900* | down | *FOXG_09438* | up | *FOXG_02035* | up |
| *FOXG_00566* | down | *FOXG_08416* | down | *FOXG_01555* | up | *FOXG_00204* | up |
| *FOXG_07963* | down | *FOXG_00945* | down | *FOXG_06295* | up | *FOXG_03016* | up |
| *FOXG_09943* | down | *FOXG_07760* | down | *FOXG_07463* | up | *FOXG_08265* | up |
| *FOXG_03178* | down | *FOXG_03471* | down | *FOXG_00571* | up | *FOXG_01603* | up |
| *FOXG_08599* | down | *FOXG_11376* | down | *FOXG_02155* | up | *FOXG_05421* | up |
| *FOXG_10337* | down | *FOXG_09179* | down | *FOXG_01064* | up | *FOXG_01190* | up |
| *FOXG_02418* | down | *FOXG_11977* | down | *FOXG_09477* | up | *FOXG_05495* | up |
| *FOXG_11536* | down | *FOXG_05287* | down | *FOXG_15405* | up | *FOXG_06351* | up |
| *FOXG_13660* | down | *FOXG_17483* | up | *FOXG_01786* | up | *FOXG_00978* | up |
| *FOXG_00814* | down | *FOXG_00946* | up | *FOXG_05135* | up | *FOXG_13807* | up |
| *FOXG_00635* | down | *FOXG_00394* | up | *FOXG_00252* | up | *FOXG_08092* | up |
| *FOXG_08699* | down | *FOXG_08495* | up | *FOXG_01883* | up | *FOXG_12883* | up |
| *FOXG_05426* | down | *FOXG_08546* | up | *FOXG_02159* | up | *FOXG_00864* | up |
| *FOXG_04703* | down | *FOXG_05976* | up | *FOXG_01697* | up | *FOXG_11545* | up |
| *FOXG_00811* | down | *FOXG_03362* | up | *FOXG_00791* | up | *FOXG_11986* | up |
| *FOXG_08390* | down | *FOXG_01531* | up | *FOXG_01751* | up | *FOXG_19785* | up |

Table S3. The information of COG.

| **Category** | **Type** | **Functional description** |
| --- | --- | --- |
| Information storage and processing | A | RNA processing and modification |
| Information storage and processing | B | Chromatin structure and dynamics |
| Information storage and processing | J | Translation, ribosomal structure and biogenesis |
| Information storage and processing | K | Transcription |
| Information storage and processing | L | Replication, recombination and repair |
| Metabolism | C | Energy production and conversion |
| Metabolism | P | Inorganic ion transport and metabolism |
| Metabolism | E | Amino acid transport and metabolism |
| Metabolism | F | Nucleotide transport and metabolism |
| Metabolism | G | Carbohydrate transport and metabolism |
| Metabolism | H | Coenzyme transport and metabolism |
| Metabolism | I | Lipid transport and metabolism |
| Metabolism | Q | Secondary metabolites biosynthesis, transport and catabolism |
| Cellular processes and signaling | D | Cell cycle control, cell division, chromosome partitioning |
| Cellular processes and signaling | M | Cell wall/membrane/envelope biogenesis |
| Cellular processes and signaling | O | Posttranslational modification, protein turnover, chaperones |
| Cellular processes and signaling | T | Signal transduction mechanisms |
| Cellular processes and signaling | U | Intracellular trafficking, secretion, and vesicular transport |
| Cellular processes and signaling | V | Defense mechanisms |
| Cellular processes and signaling | Z | Cytoskeleton |

Table S4. The accession IDs and KO names of corresponding protein.

| **Accession** | **KO name** |
| --- | --- |
| XP_018251330.1 | NOP58 |
| XP_018238894.1 | NOP1 |
| XP_018246754.1 | EIF6 |
| XP_018233589.1 | NAT10 |
| XP_018244158.1 | EMG1 |
| XP_018235519.1 | UTP10 |
| XP_018237566.1 | CSNK2B-1 |
| XP_018240584.1 | MPP10 |
| XP_018240812.1 | IMP4 |
| XP_018231726.1 | CSNK2B-2 |
| XP_018244925.1 | RIOK2 |

Table S5. Differential alternative splicing events in CK_VS_MIC.

|  | **Number of differentially expressed novel AS** | **Number of differentially expressed novel AS (*P*<0.05)** |
| --- | --- | --- |
| SE | 1167 | 4 |
| MEX | 126 | 15 |
| RI | 235 | 36 |

Table S6. Differential alternative splicing events in CK_VS_8MIC.

|  | **Number of differentially expressed novel AS** | **Number of differentially expressed novel AS (*P*<0.05)** |
| --- | --- | --- |
| SE | 1085 | 13 |
| MEX | 112 | 11 |
| RI | 207 | 36 |

Table S7. Differentially expressed proteins and significant differentially expressed proteins in CK_VS_MIC and CK_VS_8MIC.

|  | **Number of differentially expressed proteins** | **Number of significant differentially expressed proteins** |
| --- | --- | --- |
| CK_VS_MIC | 303 | 241 |
| CK_VS_8MIC | 277 | 159 |

Table S8. The information of differentially expressed genes by 11 TFs controlled in CK_VS_MIC.

| **Gene name** | **Gene description** | **TF name** |
| --- | --- | --- |
| *FOXG_08353* | DNA-binding protein creA | cre-1 |
| *FOXG_08613* | hypothetical protein | GZF3 |
| *FOXG_08613* | hypothetical protein | DAL80 |
| *FOXG_05502* | hypothetical protein | CAT8 |
| *FOXG_05502* | hypothetical protein | SIP4 |
| *FOXG_10305* | hypothetical protein | UPC2 |
| *FOXG_03084* | hypothetical protein | NCU02182 |
| *FOXG_03472* | hypothetical protein | UME6 |
| *FOXG_09750* | hypothetical protein | UPC2 |
| *FOXG_13107* | hypothetical protein | UPC2 |
| *FOXG_21153* | hypothetical protein | YNR063W |
| *FOXG_22765* | hypothetical protein | STB4 |
| *FOXG_03836* | hypothetical protein | LYS14 |

Table S9. The information of differentially expressed genes by 13 TFs controlled in CK_VS_8MIC.

| **Gene name** | **Gene description** | **TF name** |
| --- | --- | --- |
| *FOXG_03084* | hypothetical protein | NCU02182 |
| *FOXG_03472* | hypothetical protein | UME6 |
| *FOXG_11778* | hypothetical protein | RSC30 |
| *FOXG_11778* | hypothetical protein | RSC3 |
| *FOXG_09750* | hypothetical protein | UPC2 |
| *FOXG_21153* | hypothetical protein | YNR063W |
| *FOXG_16800* | hypothetical protein | nit-4 |
| *FOXG_15027* | hypothetical protein | HSF1 |
| *FOXG_15027* | hypothetical protein | SKN7 |
| *FOXG_15027* | hypothetical protein | SFL1 |
| *FOXG_15027* | hypothetical protein | HSF1 |
| *FOXG_07900* | hypothetical protein | ROX1 |
| *FOXG_03836* | hypothetical protein | LYS14 |

Table S10. The common TFs and its controlled common genes between CK_VS_MIC and CK_VS_8MIC.

| **TF name** | **Class** | **Gene_name** |
| --- | --- | --- |
| NCU02182 | Tryptophan cluster factors | *FOXG_03084* |
| UME6 | C6 zinc cluster factors | *FOXG_03472* |
| UPC2 | C6 zinc cluster factors | *FOXG_09750* |
| YNR063W | C6 zinc cluster factors | *FOXG_21153* |
| LYS14 | C6 zinc cluster factors | *FOXG_03836* |

Table S11. The information of the differentially expressed 3 TFs.

| **Accession ID** | **Description** |
| --- | --- |
| XP_018232942.1 | nuclear transcription factor Y, alpha |
| XP_018235633.1 | pH-response transcription factor pacC/RIM101 |
| XP_018256011.1 | transcription factor IWS1 |

Table S12. Gene and primers used in RT-qPCR validation.

| **Gene name** | **F** | **R** |
| --- | --- | --- |
| *FOXG_09470* | 5'-CAACCGTAAGGGTCTTCTCG-3' | 5'-CGATACGCTGGATACGGACT-3' |
| *FOXG_08276* | 5'-ATCAAACGGCTCAACATAGTGG-3' | 5'-AAGCAGACCGCCTGATACCT-3' |
| *FOXG_03084* | 5'-AGCAAACCGAGACGGAAGT-3' | 5'-ACGTCCTGAACCGATCCTTA-3' |
| *FOXG_03472* | 5'-GAAATGCCGAGAACACCCT-3' | 5'-CTCACTAATGACTTGGACGCTG-3' |
| *FOXG_09750* | 5'-AACGGCAACTCACCTCATAGC-3' | 5'-TCCCAACCGAAACAGTCCA-3' |
| *FOXG_21153* | 5'-ACGCAGAGTTTAGCAGTGGC-3' | 5'-TCTGATTCGGGTATGTCTCGT-3' |
| *FOXG_03836* | 5'-CCACCCAACCATTCACGAT-3' | 5'-CTGGCAGAAAGAAGATGAGGAG-3' |

Table S13. GO functional annotation of 4 DEGs.

| **Gene_name** | **GO term** | **GO ID** | **Description** |
| --- | --- | --- | --- |
| *FOXG_03084* | molecular_function | GO:0003677 | DNA binding |
|  |  |  |  |
| *FOXG_03472* | biological_process | GO:0006357 | regulation of transcription from RNA polymerase II promoter |
|  | cellular_component | GO:0005634 | nucleus |
|  | molecular_function | GO:0000981 | RNA polymerase II transcription factor activity, sequence-specific DNA binding |
|  |  | GO:0008270 | zinc ion binding |
|  |  |  |  |
| *FOXG_09750* | biological_process | GO:0006357 | regulation of transcription from RNA polymerase II promoter |
|  | cellular_component | GO:0005634 | nucleus |
|  | molecular_function | GO:0000981 | RNA polymerase II transcription factor activity, sequence-specific DNA binding |
|  |  | GO:0008270 | zinc ion binding |
|  |  |  |  |
| *FOXG_21153* | biological_process | GO:0006357 | regulation of transcription from RNA polymerase II promoter |
|  | cellular_component | GO:0005634 | nucleus |
|  | molecular_function | GO:0000981 | RNA polymerase II transcription factor activity, sequence-specific DNA binding |
|  |  | GO:0008270 | zinc ion binding |
|  |  |  |  |
| *FOXG_03836* | biological_process | GO:0006357 | regulation of transcription from RNA polymerase II promoter |
|  | cellular_component | GO:0005634 | nucleus |
|  | molecular_function | GO:0000981 | RNA polymerase II transcription factor activity, sequence-specific DNA binding |
|  |  | GO:0008270 | zinc ion binding |

Table S14. Detailed interactions of myriocin-NFYA/RIOK2.

| **Small molecule - amino acid residue** | **Distance** | **Donding type** |
| --- | --- | --- |
| myriocin:N7 - NFYA:GLU90:OE2 | 5.54016 | Electrostatic |
| myriocin:O6 - NFYA:ARG253:HH11 | 1.8708 | Hydrogen Bond |
| myriocin:H62 - NFYA:GLU90:OE1 | 3.07215 | Hydrogen Bond |
| myriocin:H63 - NFYA:GLU90:OE1 | 3.09328 | Hydrogen Bond |
| myriocin:H65 - NFYA:GLU90:OE1 | 2.74473 | Hydrogen Bond |
| myriocin:O2 - NFYA:ARG253:CD | 3.29141 | Hydrogen Bond |
| myriocin:C28 - NFYA:PRO119 | 5.20429 | Hydrophobic |
| myriocin:O5 - RIOK2:LYS105:HN | 2.59768 | Hydrogen Bond |
| myriocin:H62 - RIOK2:ASP246:OD1 | 2.49474 | Hydrogen Bond |
| myriocin:O5 - RIOK2:GLY104:CA | 2.71375 | Hydrogen Bond |
| myriocin:O6 - RIOK2:LEU190:CA | 3.7596 | Hydrogen Bond |
| myriocin:C28 - RIOK2:PHE232 | 3.692 | Hydrophobic |
| myriocin:C28 - RIOK2:PRO195 | 3.53468 | Hydrophobic |
| myriocin:C28 - RIOK2:ILE235 | 5.06142 | Hydrophobic |
